# Differential allele loss on chromosome 9q22.3 in human non-melanoma skin cancer.

**DOI:** 10.1038/bjc.1996.345

**Published:** 1996-07

**Authors:** E. Holmberg, B. L. Rozell, R. Toftgård

**Affiliations:** Department of Bioscience, Karolinska Institute, Huddinge, Sweden.

## Abstract

Familial predisposition to basal cell carcinoma (BCC) and squamous cell carcinoma (SCC) of the skin are apparent in the autosomal dominant syndromes naevoid basal cell carcinoma syndrome (NBCCS) and multiple self-healing squamous epitheliomata (MSSE) respectively. The gene responsible for NBCCS has been proposed to be a tumour-suppressor gene and is mapped to the same 2 Mb interval on 9q22.3 as the MSSE gene ESS1. In an attempt to further map the NBCCS gene, we have examined loss of heterozygosity (LOH) in 16 sporadic BCCs and two familial BCCs using microsatellite markers located within the candidate gene region. The overall frequency of LOH observed was 67% in the BCCs and partial or interstitial deletions were found in eight tumours, with the highest LOH frequency at markers D9S280, D9S287 and D9S180. To determine if the same genomic region also shows frequent LOH in tumours with a squamous phenotype, we have examined 11 SCCs, four actinic keratoses and 13 cases of Bowen's disease for LOH at 9q22.3. An overall LOH frequency of 50% was observed at D9S180, and occurred in all types of squamous tumours. In contrast, a much lower LOH frequency of only 6% was found at the D9S287 locus. Our observation of different patterns of LOH at 9q22.3 in sporadic BCCs and SCCs implies that more than one tumour-suppressor gene might be located in this genomic region.


British Journal of Cancer (1996) 74, 246-250
© 1996 Stockton Press All rights reserved 0007-0920/96 $12.00

### Differential allele loss on chromosome 9q22.3 in human non-melanoma skin cancer

E Holmberg[1], B L Rozell[2] and R Toftgård[1]

[1]*Department of Bioscience and Center for Nutrition and Toxicology, Karolinska Institute, Novum, S-141 57 Huddinge, Sweden,* [2]*Department of Pathology, Huddinge University Hospital, S-141 86 Huddinge, Sweden.*

**Summary** Familial predisposition to basal cell carcinoma (BCC) and squamous cell carcinoma (SCC) of the skin are apparent in the autosomal dominant syndromes naevoid basal cell carcinoma syndrome (NBCCS) and multiple self-healing squamous epitheliomata (MSSE) respectively. The gene responsible for NBCCS has been proposed to be a tumour-suppressor gene and is mapped to the same 2 Mb interval on 9q22.3 as the MSSE gene *ESS1*. In an attempt to further map the *NBCCS* gene, we have examined loss of heterozygosity (LOH) in 16 sporadic BCCs and two familial BCCs using microsatellite markers located within the candidate gene region. The overall frequency of LOH observed was 67% in the BCCs and partial or interstitial deletions were found in eight tumours, with the highest LOH frequency at markers D9S280, D9S287 and D9S180. To determine if the same genomic region also shows frequent LOH in tumours with a squamous phenotype, we have examined 11 SCCs, four actinic keratoses and 13 cases of Bowen's disease for LOH at 9q22.3. An overall LOH frequency of 50% was observed at D9S180, and occurred in all types of squamous tumours. In contrast, a much lower LOH frequency of only 6% was found at the D9S287 locus. Our observation of different patterns of LOH at 9q22.3 in sporadic BCCs and SCCs implies that more than one tumour-suppressor gene might be located in this genomic region.

**Keywords:** chromosome 9q; basal cell carcinoma; squamous cell carcinoma; naevoid basal cell carcinoma syndrome; loss of heterozygosity; microsatellite marker

Skin cancer is the most common cancer in the Western world (Ko *et al.*, 1994). A major part of these are non-melanoma skin cancers with basal cell carcinomas (BCCs) accounting for more than 75% and squamous cell carcinomas (SCCs) for 20% (Miller, 1991; Kwa *et al.*, 1992). Both BCCs and SCCs are derived from the same cell type, the keratinocyte, and show clear biological and clinical differences. BCCs grow slowly and are mainly locally aggressive, but rarely metastasise, whereas SCCs grow faster and do metastasise (Weedon, 1992). Basal cell carcinoma occurs predominantly as sporadic cases but also as familial cases in an autosomal dominant disease called the naevoid basal cell carcinoma syndrome (NBCCS) (McKusick number 109400), having an estimated minimum incidence of 1 in 55 600 (Evans *et al.*, 1993). NBCCS predisposes to multiple BCCs, and some other types of tumours such as ovarian fibromas, medulloblastomas and cardiac fibromas. The disease is also associated with widespread developmental defects (Gorlin, 1987). Squamous cell carcinoma on the other hand occurs almost exclusively as sporadic cases, although in a rare autosomal dominant disease called multiple self-healing squamous epitheliomata (MSSE) (McKusick number 132800) tumours morphologically similar to well-differentiated SCCs are seen (Goudie *et al.*, 1993). Through linkage analysis of kindreds with NBCCS the gene for this syndrome has been mapped to the 9q22-31 region (Farndon *et al.*, 1992; Gailani *et al.*, 1992; Reis *et al.*, 1992; Chenevix-Trench *et al.*, 1993). Further mapping has limited the candidate genomic region to a 2 Mb interval on 9q22.3 between microsatellite markers D9S196 and D9S180 (Goldstein *et al.*, 1994; Wicking *et al.*, 1994), whereas the interval between markers D9S12/D9S151 and D9S287 was implicated in another study (Farndon *et al.*, 1994). With regard to the disorder MSSE the corresponding gene, *ESS1*, has also been mapped to chromosome 9q22-31 (Goudie *et al.*, 1993), with a likely location in the interval between D9S196 and D9S180 (Povey *et al.*, 1994).

Correspondence: R Toftgård, Department of Bioscience, Karolinska Institute, NOVUM, S-141 57 Huddinge, Sweden
Received 23 October 1995; revised 26 January 1996; accepted 14 February 1996

The *NBCCS* gene has been proposed to be a tumour-suppressor gene based on the finding of frequent loss of heterozygosity (LOH) in the NBCCS genomic region, 9q22-31, in both sporadic and familiar BCCs, and in other tumour types seen in the syndrome such as medulloblastomas (Gailani *et al.*, 1992; Quinn *et al.*, 1994*a*; Schofield *et al.*, 1995). Further evidence that supports the tumour-suppressor gene theory is that the allele deleted in BCCs from NBCCS patients was found to be the non-disease-transmitting allele (Bonifas *et al.*, 1994). As the *NBCCS* and the *ESS1* genes map to the same genomic region it has been speculated that either two different genes reside in this area or that different mutations in the same gene may explain the different clinical features of the two disorders. Although there have not been any studies of LOH on chromosome 9q in tumours from MSSE patients, loss of chromosome 9q alleles have been reported in sporadic skin SCCs (Quinn *et al.*, 1994*b*; Zaphiropoulos *et al.*, 1994).

In order to confirm the frequent loss of the 9q22.3 area by LOH in BCCs and to further map the *NBCCS* gene region, we have examined sporadic and familiar BCCs for the occurrence of LOH using microsatellite markers located within the candidate area. To test the hypothesis that a gene in the same chromosomal area may be involved in the development of the squamous type of skin tumours we have also investigated the frequency of 9q22.3 loss in SCCs, and in the premalignant skin lesions actinic keratoses and Bowen's disease.

### Materials and methods

### *Tissue samples and DNA isolation*

Sixteen sporadic BCCs, two BCCs from a patient with NBCCS, 11 SCCs, four actinic keratoses, and 13 cases of Bowen's disease were studied. Actinic keratoses are, often multiple, sun-induced skin lesions that, if left untreated, can progress to SCCs. Bowen's disease is SCC *in situ* of the skin, with 8% of untreated cases developing into invasive SCCs (Weedon, 1992). The tumour material and matched normal tissue were selected from archival specimens from the Department of Pathology at Huddinge University Hospital.

Genomic DNA was extracted from paraffin sections of trimmed tumours judged to contain ≥50% tumour cells and from matched normal tissue. A 150 $\mu$m section of each sample was used for DNA extraction, performed according to method described elsewhere (Ma *et al.*, 1994) using Nucleon (Scotlab Ltd, Strathclyde, UK) with the following modifications. Before DNA extraction paraffin was removed by adding 100 $\mu$l of NIB buffer (0.45% NP40, 0.45% TWEEN 20, 50 mM potassium chloride, 10 mM Tris pH 8.3, 1.5 mM magnesium chloride and 100 $\mu$g ml$^{-1}$ gelatine) to the sample and boiling for 20 min followed by a 10 min centrifugation at 13 000 r.p.m. The supernatant was removed and used for further processing.

*Detection of loss of heterozygosity*

Four microsatellite markers from the putative *NBCCS/ESS1* area on chromosome 9q22.3 (D9S196, D9S280, D9S287, D9S180) were used to genotype the tumours and corresponding normal tissue using the polymerase chain reaction (PCR).

| Peak/lane | Size | Peak height | Peak area |
|---|---|---|---|
| 5B, 3 | 215.85 | 75 | 668 |
| 6B, 3 | 217.86 | 426 | 4229 |
| 7B, 3 | 218.74 | 169 | 993 |
| 8B, 3 | 219.80 | 1586 | 17033 |
| 9B, 3 | 226.27 | 244 | 2283 |
| 10B, 3 | 227.33 | 71 | 281 |
| 11B, 3 | 228.47 | 683 | 7330 |
| 9B, 4 | 215.91 | 168 | 1914 |
| 10B, 4 | 217.84 | 398 | 4202 |
| 11B, 4 | 219.87 | 1052 | 10161 |
| 12B, 4 | 224,11 | 95 | 786 |
| 13B, 4 | 226.27 | 479 | 4955 |
| 14B, 4 | 228.38 | 1200 | 12701 |

**Figure 1** Analysis of loss of heterozygosity (LOH) at the D9S180 microsatellite locus in a basal cell carcinoma (BCC), tumour no. 4. Allele sizes are 220 and 228 bp. The *x*-axis denotes size in base pairs and the *y*-axis peak height values. The table shows the peak size (in base pairs), peak height and peak area (arbitrary units). In the tumour, lane 3, there is a loss of the 228 allele resulting in an allele ratio of 0.34 compared with the normal DNA, lane 4.

The amplifications were performed in a total volume of 10 $\mu l$ containing 5 pmol of primer (one primer fluorescently labelled, all primers were synthesised on an Expedite nucleic acid synthesis system (Millipore AB, Sundbyberg, Sweden), 0.75 units *Taq* DNA Polymerase (Promega, Scandinavian Diagnostic Services, Falkenberg, Sweden), 10 mM Tris pH 9.0, 50 mM potassium chloride, 0.1% Triton X-100, 2.0 mM magnesium chloride, 200 $\mu$M dNTPs and 70–150 ng of sample DNA. This was overlaid with mineral oil. Each sample was amplified with annealing temperatures of 61°C (D9S180), 63°C (D9S287), 66°C (D9S280) and 59°C (D9S196) for 1 min in a DNA Thermal Cycler 480 (Perkin Elmer AB, Sundbyberg, Sweden). Denaturation and extension temperatures of 94°C and 72°C for 1 min each were used for all reactions, with a final 10 min extension at 72°C. The cycle number was optimised for each DNA sample to ensure that the PCR products were detectable but not overamplified. The sequences of the primers were as listed (Gyapay *et al.*, 1994), except for D9S287, which were as follows 5′-ATC ACA GGA TGC TCC TCA CGC and 5′-CTA ACC ACT ACA TTG TTC AAG GG and for D9S280 primer sequences were 5′-TCT TTT TCG CTT CCC ACC CA and 5′-CAC GCC ACT GAT CTA GGC T. The PCR products were analysed on 6% polyacrylamide denaturing gels in 1 × TBE buffer in an ABI 373A automated DNA sequencer (Applied Biosystems, Foster City, CA, USA), and subsequently analysed by Genescan 672 (Applied Biosystems, Foster City, CA, USA) for size, peak height and peak area. The method described previously (Cawkwell *et al.*, 1993), was used to determine LOH, relating the allelic imbalance of a tumour (T1:T2) to the imbalance in normal DNA (N1:N2) using the expressions T1:T2/N1:N2, or 1/ (T1:T2/ N1:N2) if allele ratio is above 1.00. Allele ratio values less or equal to 0.60 were scored as LOH.

### Results

Eighteen BCCs, 11 SCCs, four actinic keratoses and 13 cases of Bowen's disease were examined for LOH on chromosome 9q22.3, a region to which the *NBCCS* and *ESS1* gene(s) have been located. In both syndromes a predisposition to non-melanoma skin cancer is evident. Analysis of LOH was performed using an ABI automated DNA sequencer and evaluated with the Genescan software. An example demonstrating LOH at D9S180 in a BCC (tumour 4) is shown in Figure 1. All 18 BCCs were informative at one or more loci from 9q22.3. Loss of heterozygosity of the different microsatellite markers was seen in 30–57% of the informative samples (Figure 2). Loss of heterozygosity for at least one locus in the 9q22.3 area was observed in 67% (12/18) of the BCCs. Partial or interstitial deletions in the putative *NBCCS/ESS1* gene region between D9S196 and D9S180 were clearly detected in eight tumours, and possible partial or complete deletions were found in an additional four tumours (Figure 2). One tumour showed LOH for all markers in the region. The most common deleted region was between D9S180 and D9S280, with a lower frequency at D9S196. One of the tumours, no. 8, showed loss at D9S280 only, with retained normal alleles at D9S287 and D9S196. In tumours no. 1 and no. 16 LOH was seen only at the D9S180 locus. Tumour no. 15 showed loss at D9S280, was non-informative for D9S196 and retained normal alleles at D9S287. In tumour no. 17, obtained from an NBCCS patient, the chromosome region lost is derived from the non-disease-carrying chromosome as expected for a putative tumour-suppressor gene (B. Undén, personal communication). The other tumour from this patient did not show LOH for the two informative markers (D9S180 and D9S287) in the region (data not shown). All BCCs analysed were of the solid or micronodular type, classified according to WeedoN (1992). Of the tumours with LOH 8/12 were from sun-exposed sites (head/neck), which was consistent with the proportion of all BCCs (14/18) located on sun-exposed sites. No tumour showed additional bands with any of the four microsatellite markers in the *NBCCS/ESS1* region.

Loss of heterozygosity of the microsatellite marker D9S180 was seen in 7/14 (50%) of the informative samples in all subtypes of skin cancer with a squamous phenotype, compared with the microsatellite marker D9S287 where loss was only seen in one tumour (actinic keratoses), corresponding to only 6% (1/18) of the informative samples (Table I). Two tumours, no. 13 Bowen's disease and no. 4 SCC had probably lost the whole *NBCCS/ESS1* region (Figure 3). All tumours with LOH at 9q22.3 involved loss at D9S180. Eight of 11 SCCs, 4 of 13 cases of Bowen's disease and four of four actinic keratoses were located on sun-exposed sites, mainly the head/neck region. Remaining tumours were commonly found on the chest or back regions. Tumours showing LOH were predominantly found in the head/neck region (five out of seven). However, two tumours of the Bowen's disease type located on the breast and chest respectively also showed LOH at D9S180. Extra bands indicating replication errors (RER)s were not seen in any subtype of the squamous skin tumours with the microsatellite markers used.

### Discussion

In the present study the high overall frequency, 67%, of LOH on 9q22.3 in sporadic BCCs confirm earlier reports (Gailani

**Figure 2** Pattern of loss on chromosome 9q22.3 in basal cell carcinomas (BCCs) showing loss of heterozygosity (LOH). The proportion of informative tumours showing LOH at each locus is given. Tumour no. 17 is from a patient with the naevoid basal cell carcinoma syndrome (NBCCS). ■, Loss of heterozygosity; □, no loss of heterozygosity; ▧ non-informative.

**Figure 3** Pattern of loss on chromosome 9q22.3 in squamous cell carcinomas (SCCs), Bowen's disease (BD) and actinic keratoses (AK) showing loss of heterozygosity (LOH). ■, Loss of heterozygosity; □, no loss of heterozygosity; ▧ non-informative.

**Table I** Loss of heterozygosity at chromosome 9q22.3 in squamous cell carcinomas, Bowen's disease and actinic keratoses

| *Markers* | *Squamous cell carcinoma* (n=*11*) *Cases with loss* | *Informative cases* | *Bowen's disease* (n=*13*) *Cases with loss* | *Informative cases* | *Actinic keratoses* (n=*4*) *Cases with loss* | *Informative cases* | *All tumours* (n=*28*) *Cases with loss* | *Informative cases* |
|---|---|---|---|---|---|---|---|---|
| D9S180 | 2 | 4 | 4 | 8 | 1 | 2 | 7 | 14 |
| D9S287 | 0 | 8 | 0 | 8 | 1 | 2 | 1 | 18 |

*et al*., 1992; Quinn *et al*., 1994*c*; Shanley *et al*., 1995), and strongly implicates inactivation of a tumour-suppressor gene in this genomic region as a central molecular genetic change in this type of skin cancer. This is further supported by the finding that LOH at 9q22–31 occurs in BCCs derived from a Japanese population, where only a few *ras* mutations (Liew *et al*., 1991) and no p53 mutations are found (Konishi *et al*., 1994). Additionally, LOH at 9q22–31 is a more frequent event than alterations at the p53 locus in Western populations (Ziegler *et al*., 1993; van der Riet *et al*., 1994). In fact, in one study inactivation of only one p53 allele was observed in BCCs showing LOH on 9q22–31, suggesting that p53 and the putative tumour-suppressor gene on 9q22.3 may act in a cooperative manner (van der Riet *et al*., 1994). Alternatively, it is possible that the single p53 mutations represent dominant gain of function type of mutations.

Assuming that the gene inactivated in sporadic BCCs is identical to the *NBCCS* gene, consistent with the observed loss of the non-disease-transmitting allele in the familial BCCs, our analysis of eight tumours with clear partial or interstitial deletions and four tumours with possible partial or complete deletions provide information as regards the potential location of this gene. The most commonly deleted markers were D9S280, D9S287 and D9S180 with a lower frequency at D9S196, suggesting that the gene is likely to be found in an interval encompassing the former markers but distal to D9S196. Interestingly, deletions in two tumours would favour a location distal to D9S287, whereas two other tumours indicate a location proximal to this marker with one tumour showing loss of the D9S280 marker only. These findings are not consistent with the recently suggested smallest region of overlap between markers D9S287 and D9S180 (Shanley *et al*., 1995). However, in their study the putative location was based on information derived from only two sporadic BCCs. One possible way to reconcile the pattern of LOH observed in our study and published critical recombinants placing the gene between D9S196 and D9S287 (Farndon *et al*., 1994; Wicking *et al*., 1994) is if the D9S287 marker is intragenic to the *NBCCS* gene. The only genes presently known to be located in the interval between D9S196 and D9S180 is Fanconi anaemia group C (*FACC*) and xeroderma pigmentosum complementation group A (*XPAC*), which both have been excluded as the *NBCCS* gene based on mutational analysis (Bare *et al*., 1995).

In SCCs of the skin and premalignant lesions such as actinic keratoses and Bowen's disease a strikingly different pattern of LOH was observed with a high frequency of LOH (50%) observed at D9S180 but essentially no loss (6%) at D9S287. Whether a new putative tumour-suppressor gene of importance for development of SCCs resides at or distal to D9S180, with the marker D9S287 as the proximal border of the deleted region, remains to be investigated by further deletion mapping using markers distal to D9S180. One previous study reported only a low frequency of LOH at 9q22.3 in SCCs in spite of more overall widespread genetic changes compared with BCCs (Quinn *et al*., 1994*c*). However, in a subsequent study from the same group a LOH frequency of 44% in this genomic region in actinic keratoses was observed (Rehman *et al*., 1994). It is also highly interesting to note that frequent LOH in the 9q22–34 area has been reported in squamous cell carcinomas from other tissues such as oesophagus, head and neck and bladder (Ah-See *et al*., 1994; Mori *et al*., 1994; Habuchi *et al*., 1995; Miura *et al*., 1995). Furthermore, consistent with our observation of LOH at D9S180 already in premalignant lesions, these studies also support the notion that loss of genetic material on 9q is an early event in development of squamous cell cancer, whereas loss on 9p in the region harbouring the *CDKN2* gene (also called *MTS1*) appears later.

We did not detect RERs in the tumours analysed, which is consistent with earlier reports finding microsatellite length alterations at more than one loci to be rare in non-melanoma skin cancer (Zaphiropoulos *et al*., 1994; Quinn *et al*., 1995). In one of the studies (Zaphiropoulos *et al*., 1994), however, frequent RERs were observed in SCCs at one marker, D9S109, located distally to D9S180 and therefore potentially included in the region showing frequent LOH in SCCs. Taken together, the data suggest that RERs at multiple loci is an uncommon event in skin cancer.

UV radiation is considered to be the most important aetiological factor for development of both BCCs and SCCs (Ko *et al*., 1994; Gallagher *et al*., 1995*a,b*) and a high frequency of UV-related mutations in the p53 gene, mainly C to T and CC to TT, have been found in both types of tumours (Brash *et al*., 1991; Ziegler *et al*., 1993; van der Riet *et al*., 1994). It is therefore tempting to believe that UV radiation may also be a key factor behind the genetic alterations on 9q. There are, however, several reasons to think that other, so far unknown, causative factors may play a role. Firstly, LOH on 9q was observed in both BCCs and Bowen's disease derived from sites not regularly sun exposed and secondly, LOH was found in BCCs from a Japanese population lacking UV-related p53 mutations (Konishi *et al*., 1994). Admittedly, it still remains possible that appearance of LOH is favoured as a primary event during a severe sunburn and that subsequent p53 mutations are more closely related to accumulated sun exposure.

In summary, analysis of LOH at 9q22.3 in sporadic BCCs suggest that the putative tumour-suppressor gene, most likely identical to the *NBCCS* gene, resides in a region encompassing the markers D9S280, D9S287 and D9S180 and is compatible with an intragenic location of the marker D9S287. Moreover, the different pattern of LOH in SCCs and premalignant lesions implies that loss of a putative tumour-suppressor gene important in development of the squamous type of skin cancers might be located in or distal to this area.

**Acknowledgements**
We are grateful to Dr Peter G Zaphiropoulos for technical advice. This work was supported by grants from the Swedish Cancer Fund, the Swedish Radiation Protection Institute and from Edvard Welanders Stiftelse/Finsenstiftelsen.

### References

AH-SEE KW, COOKE TG, PICKFORD IR, SOUTAR D AND BALMAIN A. (1994). An allelotype of squamous carcinoma of the head and neck using microsatellite markers. *Cancer Res*., **54**, 1617–1621.

BARE JW, XIE J, QUINN AG AND EPSTEIN EH JR. (1995). Analysis of the FACC and XPAC genes as candidate genes for the basal cell nevus syndrome. *J. Invest. Dermatol*., **104**, 604.

BONIFAS JM, BARE JW, KERSCHMANN RL, MASTER SP AND EPSTEIN, EH, JR. (1994). Parental origin of chromosome 9q22.3-q31 lost in basal cell carcinomas from basal cell nevus syndrome patients. *Hum. Mol. Genet.*, **3**, 447-448.

BRASH DE, RUDOLPH JA, SIMON JA, LIN A, McKENNA GJ, BADEN HP, HALPERIN AJ AND PONTEN J. (1991). A role for sunlight in skin cancer: UV-induced p53 mutations in squamous cell carcinoma. *Proc. Natl Acad. Sci. USA*, **88**, 10124-10128.

CAWKWELL L, BELL SM, LEWIS FA, DIXON MF, TAYLOR GR AND QUIRKE P. (1993). Rapid detection of allele loss in colorectal tumours using microsatellites and fluorescent DNA technology. *Br. J. Cancer*, **67**, 1262-1267.

CHENEVIX-TRENCH G, WICKING C, BERKMAN J, SHARPE H, HOCKEY A, HAAN E, OLEY C, RAVINE D, TURNER A, GOLDGAR D, SEARLE J AND WAINWRIGHT B. (1993). Further localization of the gene for nevoid basal cell carcinoma syndrome (NBCCS) in 15 Australasian families: linkage and loss of heterozygosity. *Am. J. Hum. Genet.*, **53**, 760-767.

EVANS DGR, LADUSANS EJ, RIMMER S, BURNELL LD, THAKKER N AND FARNDON PA. (1993). Complications of the naevoid basal cell carcinoma syndrome: results of a population based study. *J. Med. Genet.*, **30**, 460-464.

FARNDON PA, DEL MASTRO RG, EVANS DGR AND KILPATRICK MW. (1992). Location of gene for Gorlin syndrome. *Lancet*, **339**, 581-582.

FARNDON PA, MORRIS DJ, HARDY C, McCONVILLE CM, WEISSENBACH J, KILPATRICK MW AND REIS A. (1994). Analysis of 133 meioses places the genes for nevoid basal cell carcinoma (Gorlin) syndrome and Fanconi anemia group C in a 2.6-cM interval and contributes to the fine map of 9q22.3. *Genomics*, **23**, 486-489.

GAILANI MR, BALE SJ, LEFFELL DJ, DiGIOVANNA JJ, PECK GL, POLIAK S, DRUM MA, PASTAKIA B, McBRIDE OW, KASE R, GREENE M, MULVIHILL JJ AND BALE AE. (1992). Developmental defects in Gorlin syndrome related to a putative tumor suppressor gene on chromosome 9. *Cell*, **69**, 111-117.

GALLAGHER RP, HILL GB, BAJDIK CD, FINCHAM S, COLDMAN AJ, McLEAN DI AND THRELFALL WJ. (1995*a*). Sunlight exposure, pigmentary factors, and risk of nonmelanocytic skin cancer. I. Basal cell carcinoma. *Arch. Dermatol.*, **131**, 157-163.

GALLAGHER RP, HILL GB, BAJDIK CD, COLDMAN AJ, FINCHAM S, McLEAN DI AND THRELFALL WJ. (1995*b*). Sunlight exposure, pigmentation factors, and risk of nonmelanocytic skin cancer. II. Squamous cell carcinoma. *Arch. Dermatol.*, **131**, 164-169.

GOLDSTEIN AM, STEWART C, BALE AE, BALE SJ AND DEAN M. (1994). Localization of the gene for the nevoid basal cell carcinoma syndrome. *Am. J. Hum. Genet.*, **54**, 765-773.

GORLIN RJ. (1987). Nevoid basal cell carcinoma syndrome. *Medicine*, **66**, 98-113.

GOUDIE DR, YUILLE MA, LEVERSHA MA, FURLONG RA, CARTER NP, LUSH MJ, AFFARA NA AND FERGUSON-SMITH MA. (1993). Multiple self-healing squamous epitheliomata (ESS1) mapped to chromosome 9q22-q31 in families with common ancestry. *Nature Genet.*, **3**, 165-169.

GYAPAY G, MORISETTE J, VIGNAL A, DIB C, FIZAMES C, MILLASSEAU P, MARC S, BENARDI G, LATHROP M AND WEISSENBACH J. (1994). The 1993-94 Généthon human genetic linkage map. *Nature Genet.*, **7**, 246-339.

HABUCHI T, DEVLIN J, ELDER PA AND KNOWLES MA. (1995). Detailed deletion mapping of chromosome 9q in bladder cancer: evidence for two tumour suppressor loci. *Oncogene*, **11**, 1671-1674.

KO CB, WALTON S, KECZKES K, BURY HPR AND NICHOLSON C. (1994). The emerging epidemic of skin cancer. *Br. J. Dermatol.*, **130**, 269-272.

KONISHI K, YAMANISHI K, ISHIZAKI K, YAMADA K, KISHIMOTO S AND YASUNO H. (1994). Analysis of p53 gene mutations and loss of heterozygosity for loci on chromosome 9q in basal cell carcinoma. *Cancer Lett.*, **79**, 67-72.

KWA RE, CAMPANA K AND MOY RL. (1992). Biology of cutaneous squamous cell carcinoma (Review). *J. Am. Acad. Dermatol.*, **26**, 1-26.

LIEW FM, YAMANISHI K, KONISHI K, KISHIMOTO S AND YASUNO H. (1991). Low incidence of Ha-*ras* oncogene mutations in human epidermal tumors. *Cancer Lett.*, **59**, 231-235.

MA HW, CHENG J AND CADDY B. (1994). Extraction of high quality genomic DNA from microsamples of human blood. *J. Forensic Sci. Soc.*, **34**, 231-235.

MILLER SJ. (1991). Biology of basal cell carcinoma (Part I) (Review). *J. Am. Acad. Dermatol.*, **24**, 1-13.

MIURA K, OKITA K, FURUKAWA Y, MATSUNO S AND NAKAMURA Y. (1995). Deletion mapping in squamous cell carcinomas of the esophagus defines a region containing a tumor suppressor gene within a 4-centimorgan interval of the distal long arm of chromosome 9. *Cancer Res.*, **55**, 1828-1830.

MORI T, YANAGISAWA A, KATO Y, MIURA K, NISHIHIRA T, MORI S AND NAKAMURA Y. (1994). Accumulation of genetic alterations during esophageal carcinogenesis. *Hum. Mol. Genet.*, **3**, 1969-1971.

POVEY S, ARMOUR J, FARNDON P, HAINES JL, KNOWLES M, OLOPADE F, PILZ A, WHITE JA AND KWIATKOWSKI DJ. (1994). Report and abstracts of the Third International Workshop on Chromosome 9. Cambridge, United Kingdom, 9-11 April, 1994. *Ann. Hum. Genet.*, **58**, 177-250.

QUINN AG, SIKKINK S AND REES JL. (1994*a*). Basal cell carcinomas and squamous cell carcinomas of human skin show distinct patterns of chromosome loss. *Cancer Res.*, **54**, 4756-4759.

QUINN AG, CAMPBELL C, HEALY E AND REES JL. (1994*b*). Chromosome 9 allele loss occurs in both basal and squamous cell carcinomas of the skin. *J. Invest. Dermatol.*, **102**, 300-303.

QUINN AG, SIKKINK S AND REES JL. (1994*c*). Delineation of two distinct deleted regions on chromosome 9 in human non-melanoma skin cancers. *Genes Chrom. Cancer*, **11**, 222-225.

QUINN AG, HEALY E, REHMAN I, SIKKINK S AND REES JL. (1995). Microsatellite instability in human non-melanoma and melanoma skin cancer. *J. Invest. Dermatol.*, **104**, 309-312.

REHMAN I, QUINN AG, HEALY E AND REES JL. (1994). High frequency of loss of heterozygosity in actinic keratoses, a usually benign disease. *Lancet*, **344**, 788-789.

REIS A, KUSTER W, GEBEL E, FUHRMANN W, GROTH W, KUKLIK M, WEGNER RD, LINSS G, HAMM H, WOLFF G, GUSTAFSON G, BÜRGER J AND NEITZEL H. (1992). Localisation of gene for the naevoid basal-cell carcinoma syndrome. *Lancet*, **339**, 617.

SCHOFIELD D, WEST DC, ANTHONY DC, MARSHAL R AND SKLAR J. (1995). Correlation of loss of heterozygosity at chromosome 9q with histological subtype in medulloblastomas. *Am. J. Pathol.*, **146**, 472-480.

SHANLEY SM, DAWKINS H, WAINWRIGHT BJ, WICKING C, HEENAN P, ELDON M, SEARLE J AND CHENEVIX-TRENCH G. (1995). Fine deletion mapping on the long arm of chromosome 9 in sporadic and familial basal cell carcinomas. *Hum. Mol. Genet.*, **4**, 129-133.

VAN DER RIET P, KARP D, FARMER E, WEI Q, GROSSMAN L, TOKINO K, RUPPERT JM AND SIDRANSKY D. (1994). Progression of basal cell carcinoma through loss of chromosome 9q and inactivation of a single p53 allele. *Cancer Res.*, **54**, 25-27.

WEEDON D. (1992). Tumours of the epidermis. In *Systemic Pathology: The Skin*, Vol. 9, 3rd edn, Symmers W. St C. (ed.) pp. 729-776. Churchill Livingstone: London.

WICKING C, BERKMAN J, WAINWRIGHT B AND CHENEVIX-TRENCH G. (1994). Fine genetic mapping of the gene for nevoid basal cell carcinoma syndrome. *Genomics*, **22**, 505-511.

ZAPHIROPOULOS PG, SÖDERKVIST P, HEDBLAD MA AND TOFTGÅRD R. (1994). Genetic instability of microsatellite markers in region q22.3-q31 of chromosome 9 in skin squamous cell carcinomas. *Biochem. Biophys. Res. Communications*, **201**, 1495-1501.

ZIEGLER A, LEFFELL DJ, KUNALA S, SHARMA HW, GAILANI M, SIMON JA, HAILPERIN AJ, BADEN HP, SHAPIRO PE, BALE AE AND BRASH DE. (1993). Mutation hotspots due to sunlight in the p53 gene of nonmelanoma skin cancers. *Proc. Natl Acad. Sci. USA*, **90**, 4216-4220.


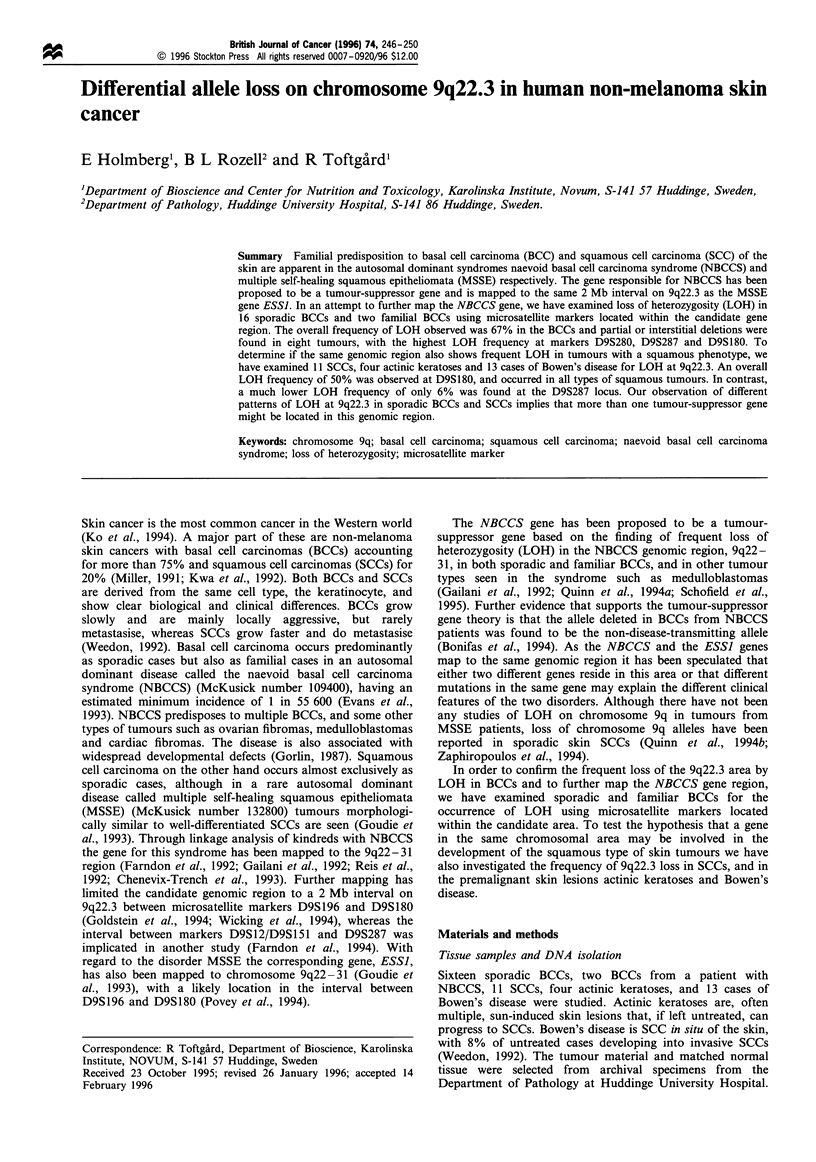

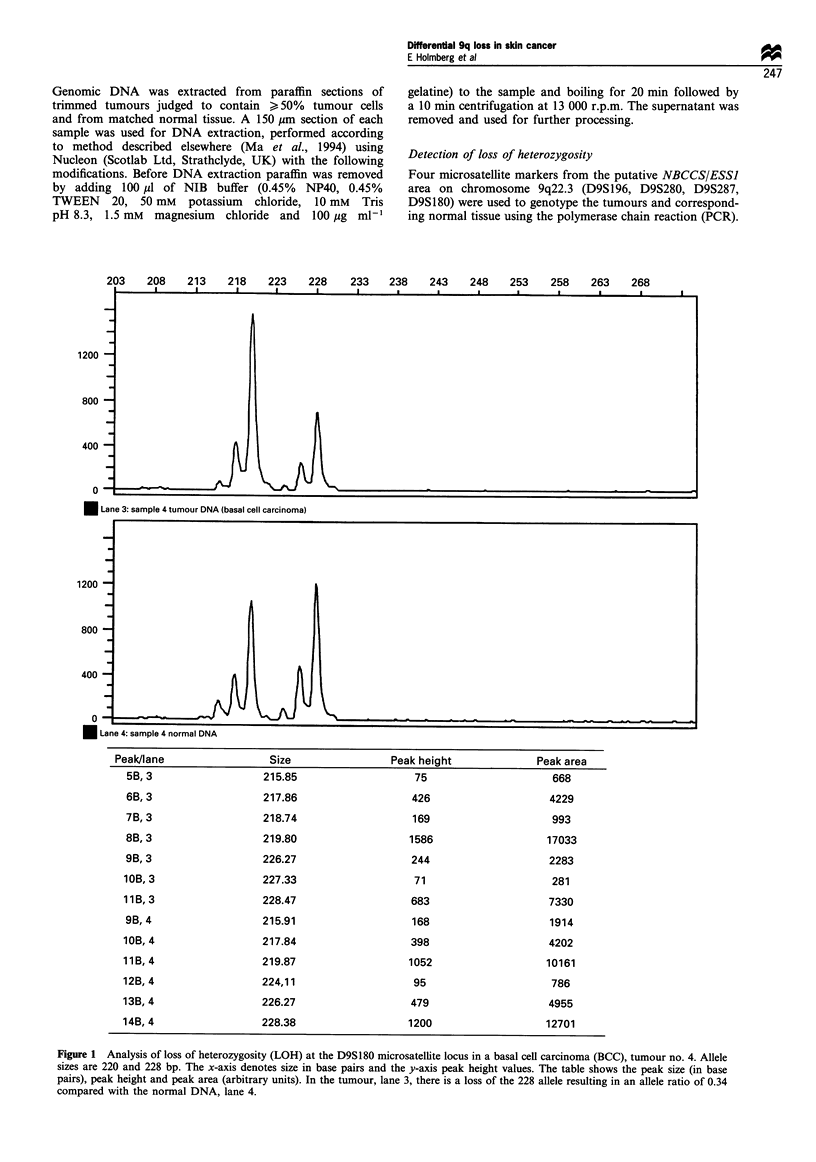

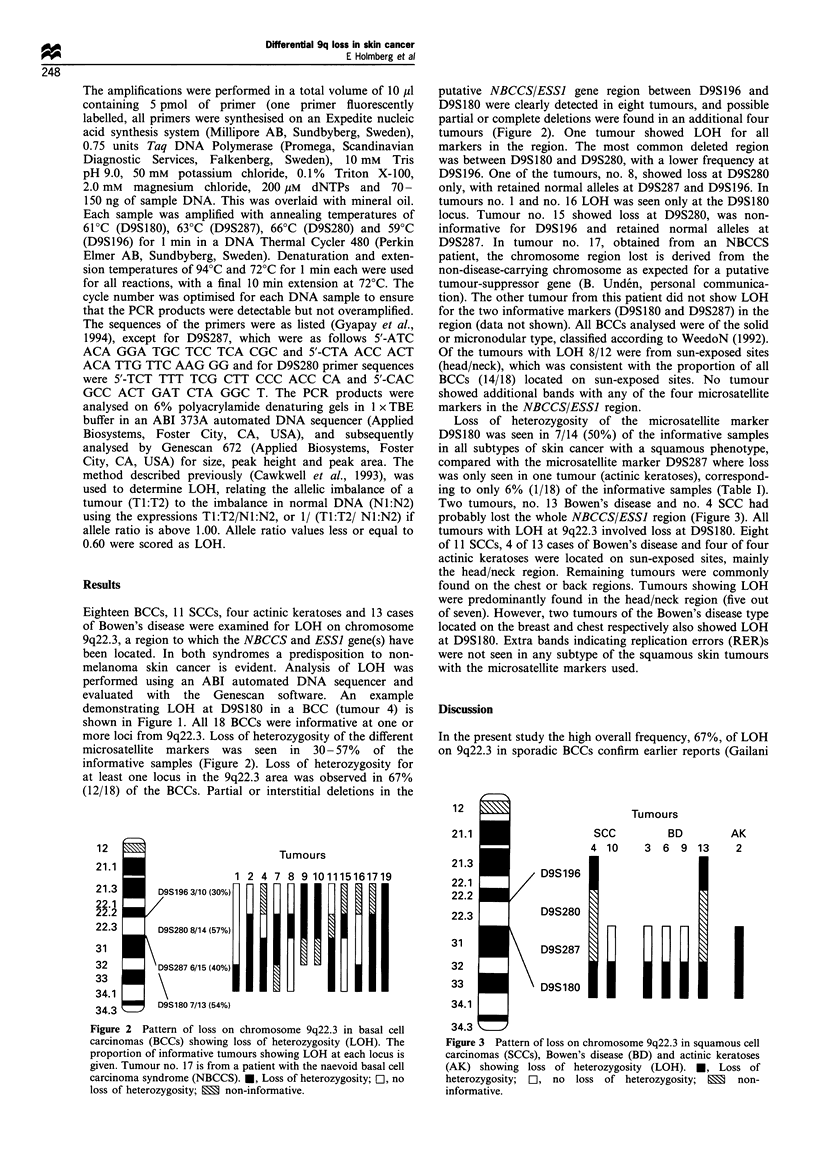

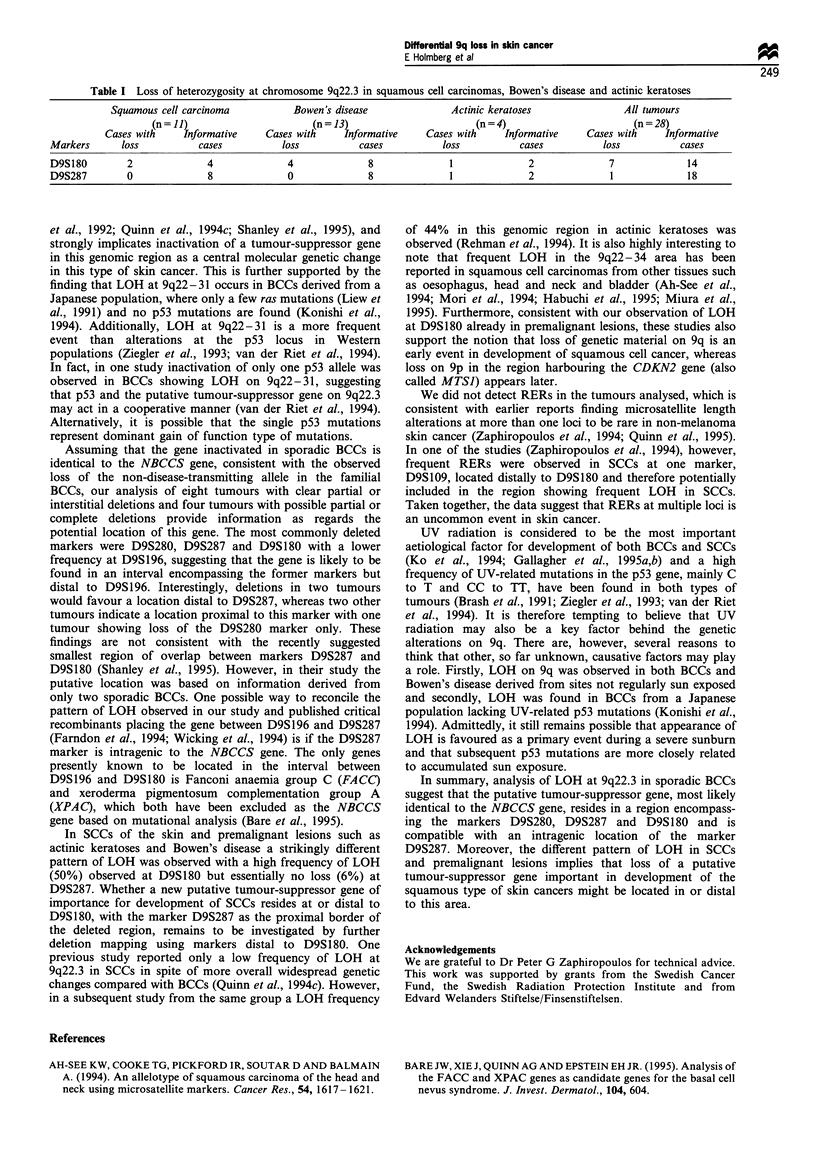

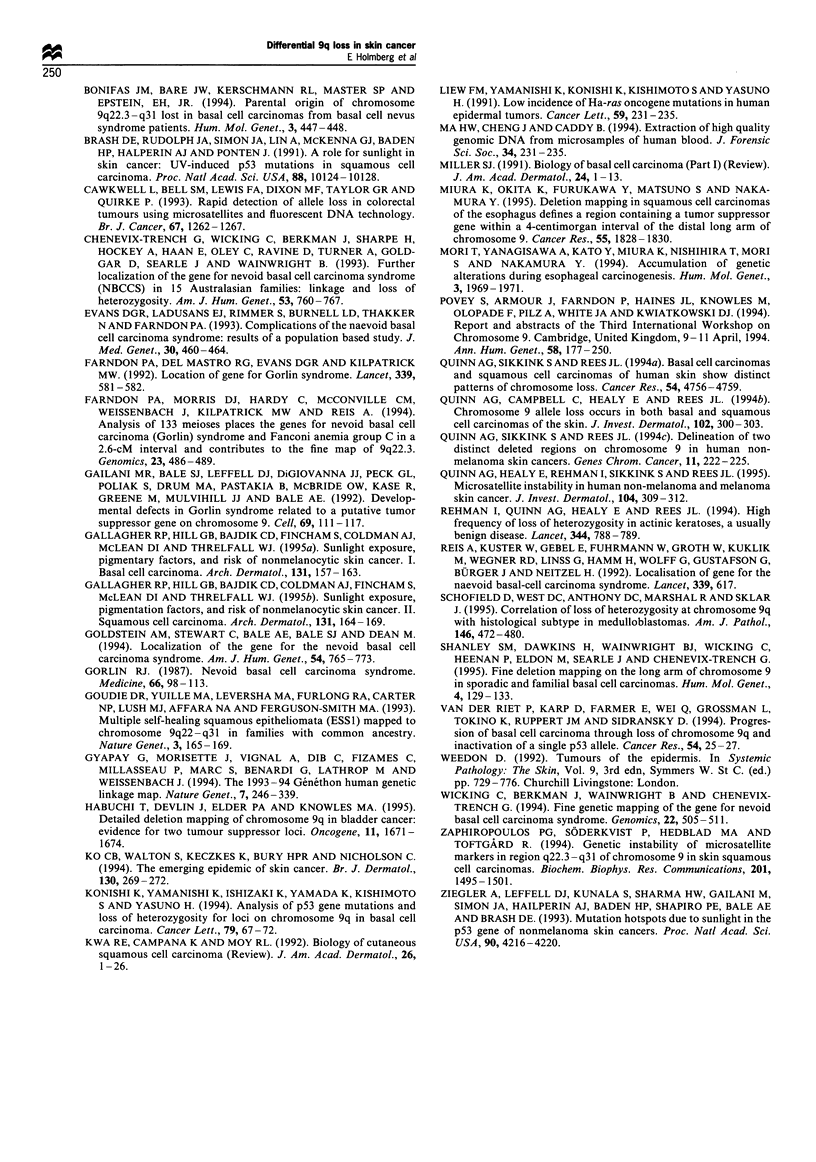


## References

[OCR_00588] Ah-See K. W., Cooke T. G., Pickford I. R., Soutar D., Balmain A. (1994). An allelotype of squamous carcinoma of the head and neck using microsatellite markers.. Cancer Res.

[OCR_00603] Bonifas J. M., Bare J. W., Kerschmann R. L., Master S. P., Epstein E. H. (1994). Parental origin of chromosome 9q22.3-q31 lost in basal cell carcinomas from basal cell nevus syndrome patients.. Hum Mol Genet.

[OCR_00610] Brash D. E., Rudolph J. A., Simon J. A., Lin A., McKenna G. J., Baden H. P., Halperin A. J., Pontén J. (1991). A role for sunlight in skin cancer: UV-induced p53 mutations in squamous cell carcinoma.. Proc Natl Acad Sci U S A.

[OCR_00613] Cawkwell L., Bell S. M., Lewis F. A., Dixon M. F., Taylor G. R., Quirke P. (1993). Rapid detection of allele loss in colorectal tumours using microsatellites and fluorescent DNA technology.. Br J Cancer.

[OCR_00619] Chenevix-Trench G., Wicking C., Berkman J., Sharpe H., Hockey A., Haan E., Oley C., Ravine D., Turner A., Goldgar D. (1993). Further localization of the gene for nevoid basal cell carcinoma syndrome (NBCCS) in 15 Australasian families: linkage and loss of heterozygosity.. Am J Hum Genet.

[OCR_00630] Evans D. G., Ladusans E. J., Rimmer S., Burnell L. D., Thakker N., Farndon P. A. (1993). Complications of the naevoid basal cell carcinoma syndrome: results of a population based study.. J Med Genet.

[OCR_00636] Farndon P. A., Del Mastro R. G., Evans D. G., Kilpatrick M. W. (1992). Location of gene for Gorlin syndrome.. Lancet.

[OCR_00641] Farndon P. A., Morris D. J., Hardy C., McConville C. M., Weissenbach J., Kilpatrick M. W., Reis A. (1994). Analysis of 133 meioses places the genes for nevoid basal cell carcinoma (Gorlin) syndrome and Fanconi anemia group C in a 2.6-cM interval and contributes to the fine map of 9q22.3.. Genomics.

[OCR_00649] Gailani M. R., Bale S. J., Leffell D. J., DiGiovanna J. J., Peck G. L., Poliak S., Drum M. A., Pastakia B., McBride O. W., Kase R. (1992). Developmental defects in Gorlin syndrome related to a putative tumor suppressor gene on chromosome 9.. Cell.

[OCR_00662] Gallagher R. P., Hill G. B., Bajdik C. D., Coldman A. J., Fincham S., McLean D. I., Threlfall W. J. (1995). Sunlight exposure, pigmentation factors, and risk of nonmelanocytic skin cancer. II. Squamous cell carcinoma.. Arch Dermatol.

[OCR_00656] Gallagher R. P., Hill G. B., Bajdik C. D., Fincham S., Coldman A. J., McLean D. I., Threlfall W. J. (1995). Sunlight exposure, pigmentary factors, and risk of nonmelanocytic skin cancer. I. Basal cell carcinoma.. Arch Dermatol.

[OCR_00665] Goldstein A. M., Stewart C., Bale A. E., Bale S. J., Dean M. (1994). Localization of the gene for the nevoid basal cell carcinoma syndrome.. Am J Hum Genet.

[OCR_00672] Gorlin R. J. (1987). Nevoid basal-cell carcinoma syndrome.. Medicine (Baltimore).

[OCR_00677] Goudie D. R., Yuille M. A., Leversha M. A., Furlong R. A., Carter N. P., Lush M. J., Affara N. A., Ferguson-Smith M. A. (1993). Multiple self-healing squamous epitheliomata (ESS1) mapped to chromosome 9q22-q31 in families with common ancestry.. Nat Genet.

[OCR_00684] Gyapay G., Morissette J., Vignal A., Dib C., Fizames C., Millasseau P., Marc S., Bernardi G., Lathrop M., Weissenbach J. (1994). The 1993-94 Généthon human genetic linkage map.. Nat Genet.

[OCR_00689] Habuchi T., Devlin J., Elder P. A., Knowles M. A. (1995). Detailed deletion mapping of chromosome 9q in bladder cancer: evidence for two tumour suppressor loci.. Oncogene.

[OCR_00693] Ko C. B., Walton S., Keczkes K., Bury H. P., Nicholson C. (1994). The emerging epidemic of skin cancer.. Br J Dermatol.

[OCR_00698] Konishi K., Yamanishi K., Ishizaki K., Yamada K., Kishimoto S., Yasuno H. (1994). Analysis of p53 gene mutations and loss of heterozygosity for loci on chromosome 9q in basal cell carcinoma.. Cancer Lett.

[OCR_00706] Kwa R. E., Campana K., Moy R. L. (1992). Biology of cutaneous squamous cell carcinoma.. J Am Acad Dermatol.

[OCR_00709] Lieu F. M., Yamanishi K., Konishi K., Kishimoto S., Yasuno H. (1991). Low incidence of Ha-ras oncogene mutations in human epidermal tumors.. Cancer Lett.

[OCR_00714] Ma H. W., Cheng J., Caddy B. (1994). Extraction of high quality genomic DNA from microsamples of human blood.. J Forensic Sci Soc.

[OCR_00721] Miller S. J. (1991). Biology of basal cell carcinoma (Part I).. J Am Acad Dermatol.

[OCR_00725] Miura K., Okita K., Furukawa Y., Matsuno S., Nakamura Y. (1995). Deletion mapping in squamous cell carcinomas of the esophagus defines a region containing a tumor suppressor gene within a 4-centimorgan interval of the distal long arm of chromosome 9.. Cancer Res.

[OCR_00732] Mori T., Yanagisawa A., Kato Y., Miura K., Nishihira T., Mori S., Nakamura Y. (1994). Accumulation of genetic alterations during esophageal carcinogenesis.. Hum Mol Genet.

[OCR_00739] Povey S., Armour J., Farndon P., Haines J. L., Knowles M., Olopade F., Pilz A., White J. A., Kwiatkowski D. J. (1994). Report and abstracts of the Third International Workshop on Chromosome 9. Cambridge, United Kingdom, 9-11 April, 1994.. Ann Hum Genet.

[OCR_00750] Quinn A. G., Campbell C., Healy E., Rees J. L. (1994). Chromosome 9 allele loss occurs in both basal and squamous cell carcinomas of the skin.. J Invest Dermatol.

[OCR_00758] Quinn A. G., Healy E., Rehman I., Sikkink S., Rees J. L. (1995). Microsatellite instability in human non-melanoma and melanoma skin cancer.. J Invest Dermatol.

[OCR_00745] Quinn A. G., Sikkink S., Rees J. L. (1994). Basal cell carcinomas and squamous cell carcinomas of human skin show distinct patterns of chromosome loss.. Cancer Res.

[OCR_00755] Quinn A. G., Sikkink S., Rees J. L. (1994). Delineation of two distinct deleted regions on chromosome 9 in human non-melanoma skin cancers.. Genes Chromosomes Cancer.

[OCR_00765] Rehman I., Quinn A. G., Healy E., Rees J. L. (1994). High frequency of loss of heterozygosity in actinic keratoses, a usually benign disease.. Lancet.

[OCR_00771] Reis A., Küster W., Linss G., Gebel E., Hamm H., Fuhrmann W., Wolff G., Groth W., Gustafson G., Kuklik M. (1992). Localisation of gene for the naevoid basal-cell carcinoma syndrome.. Lancet.

[OCR_00776] Schofield D., West D. C., Anthony D. C., Marshal R., Sklar J. (1995). Correlation of loss of heterozygosity at chromosome 9q with histological subtype in medulloblastomas.. Am J Pathol.

[OCR_00782] Shanley S. M., Dawkins H., Wainwright B. J., Wicking C., Heenan P., Eldon M., Searle J., Chenevix-Trench G. (1995). Fine deletion mapping on the long arm of chromosome 9 in sporadic and familial basal cell carcinomas.. Hum Mol Genet.

[OCR_00801] Wicking C., Berkman J., Wainwright B., Chenevix-Trench G. (1994). Fine genetic mapping of the gene for nevoid basal cell carcinoma syndrome.. Genomics.

[OCR_00803] Zaphiropoulos P. G., Söderkvist P., Hedblad M. A., Toftgård R. (1994). Genetic instability of microsatellite markers in region q22.3-q31 of chromosome 9 in skin squamous cell carcinomas.. Biochem Biophys Res Commun.

[OCR_00810] Ziegler A., Leffell D. J., Kunala S., Sharma H. W., Gailani M., Simon J. A., Halperin A. J., Baden H. P., Shapiro P. E., Bale A. E. (1993). Mutation hotspots due to sunlight in the p53 gene of nonmelanoma skin cancers.. Proc Natl Acad Sci U S A.

[OCR_00787] van der Riet P., Karp D., Farmer E., Wei Q., Grossman L., Tokino K., Ruppert J. M., Sidransky D. (1994). Progression of basal cell carcinoma through loss of chromosome 9q and inactivation of a single p53 allele.. Cancer Res.

